# Complex decision-making in pregnancy-associated infective endocarditis: a case series

**DOI:** 10.1093/ehjcr/ytag057

**Published:** 2026-01-28

**Authors:** Jordan Liebman, Emily C McQuade, Syed Hussain, Mathew Williams, Christina A Penfield, Ashley S Roman, Dan G Halpern, Adam Small

**Affiliations:** Department of Medicine, NYU Grossman School of Medicine, 550 First Avenue, New York, NY 10016, USA; Department of Medicine, NYU Grossman School of Medicine, 550 First Avenue, New York, NY 10016, USA; Department of Cardiothoracic Surgery, NYU Grossman School of Medicine, 550 First Avenue, New York, NY 10016, USA; Department of Cardiothoracic Surgery, NYU Grossman School of Medicine, 550 First Avenue, New York, NY 10016, USA; Division of Maternal-Fetal Medicine, Department of Obstetrics and Gynecology, NYU Grossman School of Medicine, 550 First Avenue, New York, NY 10016, USA; Division of Maternal-Fetal Medicine, Department of Obstetrics and Gynecology, NYU Grossman School of Medicine, 550 First Avenue, New York, NY 10016, USA; Leon H. Charney Division of Cardiology, NYU Grossman School of Medicine, 550 First Avenue, New York, NY 10016, USA; Leon H. Charney Division of Cardiology, NYU Grossman School of Medicine, 550 First Avenue, New York, NY 10016, USA

**Keywords:** Infective endocarditis, Pregnancy, Mitral valve, Cardiopulmonary bypass, Intraoperative foetal monitoring, Surgical valve replacement, Case report

## Abstract

**Background:**

Although infective endocarditis during pregnancy is rare, it carries significant morbidity and mortality for both mother and foetus. While professional societies provide recommendations for the treatment of infective endocarditis, there are no specific guidelines for the management of pregnancy-associated infective endocarditis. In this report, we present two cases of infective endocarditis presenting during the second trimester of pregnancy that required surgical intervention, focusing on the unique considerations when caring for pregnant individuals.

**Case summaries:**

Two patients in the second trimester of pregnancy presented with fevers and malaise. Both were found to have positive blood cultures and mitral valve vegetations, leading to diagnoses of mitral valve endocarditis. Their hospital courses were complicated by embolic strokes, and one patient required transcatheter embolization of a mycotic aneurysm. Both patients underwent surgical valve replacements with bioprosthetic valves. Ultimately, both patients delivered at term without complication.

**Discussion:**

In addition to the standard management of infective endocarditis, pregnancy-associated infective endocarditis requires multidisciplinary collaboration regarding the relative timing of cardiac surgery and delivery, the use of intraoperative foetal monitoring, and the choice of valve replacement and anticoagulation. Each of these decisions requires balancing the risk of morbidity and mortality to the patient, the risk of neonatal prematurity and associated complications and disability, and the risk of foetal death during cardiopulmonary bypass. We discuss our teams’ decision-making processes with a focus on the relevant considerations for each of these challenging decisions.

Learning points

- Deciding whether to pursue delivery before, during, or after non-obstetric surgery depends on the gestational age of the foetus, the risk of surgery, and the patient’s preferences.
- The decision to use intraoperative foetal monitoring during cardiac surgery depends on the type of cardiac surgery, gestational age, patient preferences, and availability of obstetric resources.
- Choosing the type of valve replacement for pregnant patients involves balancing the risk of future valve replacement and the risk of anticoagulation during pregnancy.

## Introduction

Although the incidence of infective endocarditis (IE) during pregnancy is estimated at less than 0.01%,^1^ it carries significant morbidity and mortality for mother and fetus.^1^ Risk factors for pregnancy-associated IE mirror the traditional risk factors for IE: Intravenous drug use, congenital heart disease, and rheumatic heart disease.^2^ Most infections are caused by viridans streptococci and involve the left-sided valves.^3^ In this report, we present two cases of IE during the second trimester of pregnancy requiring surgical intervention, focusing on the unique considerations when caring for pregnant individuals with IE.

## Summary figure

**Figure:**
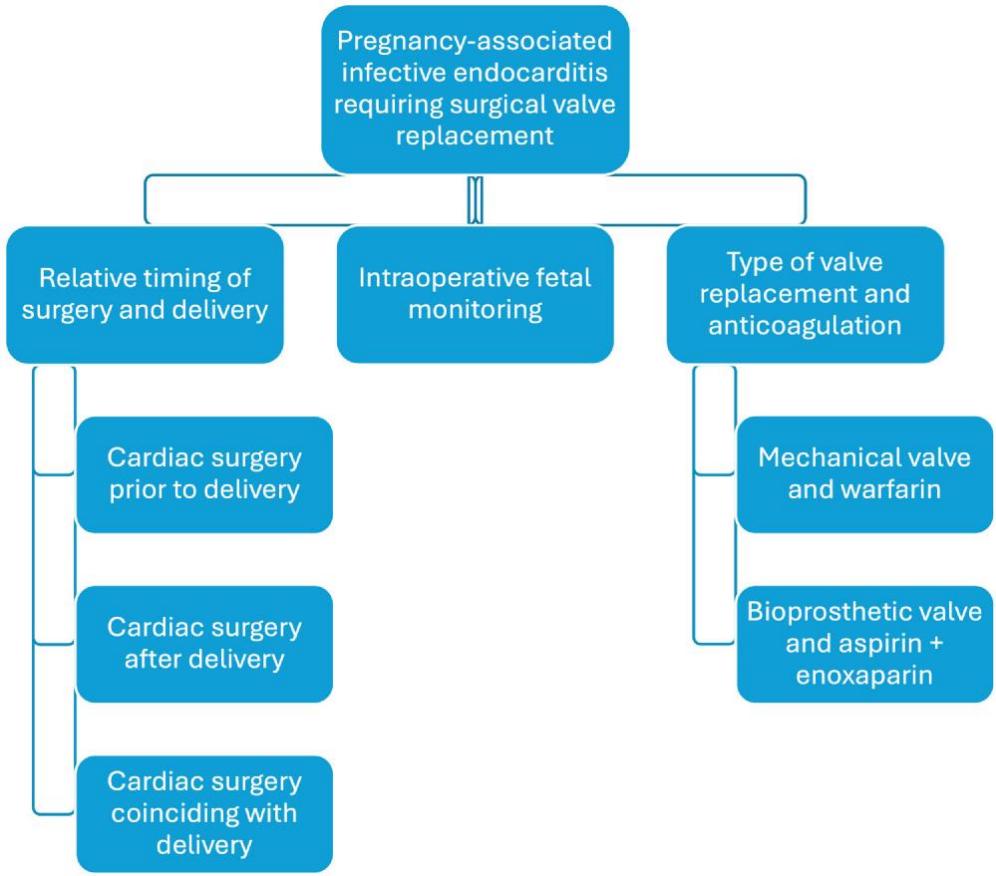


The treatment of IE during pregnancy presents several challenges related to changes in cardiovascular physiology during pregnancy and balancing the needs of the patient and foetus. During pregnancy, the cardiovascular system adapts by increasing cardiac output through vasodilation, increased heart rate, and increased plasma volume.^4^ Infection disrupts this equilibrium, causing hypoperfusion of the patient’s organs and the placenta. A systematic review of IE during pregnancy and postpartum found a maternal mortality rate of 11.2%, along with a 33% rate of pregnancy loss, 8.8% rate of maternal complications, and 10.8% rate of neonatal complications.^2^ Moreover, surgical interventions for IE, which usually require cardiopulmonary bypass (CPB), expose the foetus to significant periprocedural risk.^5–7^

While professional societies provide recommendations for the management of IE,^8–12^ there are no definitive guidelines for the management of IE during pregnancy, leaving physicians to make decisions on a case-by-case basis.^8,12,13^ Here we present two cases of IE requiring surgical intervention during the second trimester of pregnancy. Given the paucity of recommendations for this population, we focus on decision-making regarding the timing of surgical intervention relative to delivery, the use of intraoperative foetal monitoring (IFM), and choice of valve replacement.

### Patient 1

**Figure 1:**
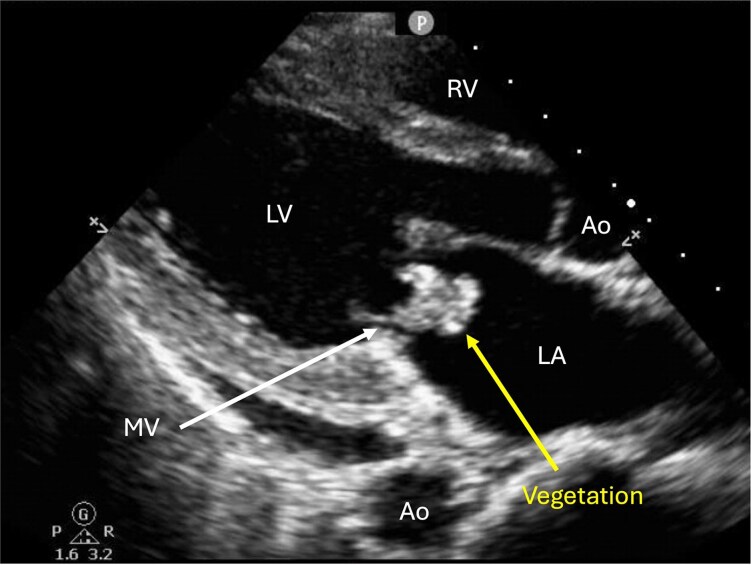
Case 1 (Mitral Valve Vegetation): transthoracic echocardiogram (TTE) in parasternal long axis. The lobulated, irregular, mobile mass seen attached to the atrial side of the mitral valve represents a vegetation, consistent with the diagnosis of infective endocarditis. This figure corresponds to Supplementary material online, *Video S1*. Ao, aorta; LA, left atrium; LV, left ventricle; MV, mitral valve; RV, right ventricle.

A 32-year-old G2P1001 female at 25-weeks’ gestation presented with fevers, malaise, and dyspnoea. She was noted to be febrile (38.8°C), tachycardic [136 beats per minute (BPM)], and hypotensive (83/52 mmHg) with a systolic murmur, lung crackles, and bilateral pitting oedema. Initial labs were notable for leucocytosis (17.9 × 10^3^/μL) with 1% bands, severe anaemia (6.4 g/dL), thrombocytopenia (13 × 10^3^/μL), and elevated troponin (214 ng/L). Blood cultures grew *Haemophilus parainfluenza*. Transthoracic echocardiogram showed moderate to severe mitral regurgitation with a 2.5 cm vegetation on the mitral valve (MV; *Figure 1*; Supplementary material online, *Videos S1* and *S2*). She was diagnosed with IE and heart failure, admitted to the intensive care unit, and given vancomycin (1 g every 8 h), piperacillin-tazobactam (4.5 g every 8 h), and several doses of intravenous furosemide (10 mg) with blood transfusions. Betamethasone (12 g given twice, 24 h apart) was administered to promote foetal lung maturity. Anaemia and thrombocytopenia were attributed to bone marrow suppression from systemic infection and managed with blood transfusions and romiplostim.

On the second day of her hospitalization, she developed new left arm numbness and weakness. Magnetic resonance imaging (MRI) of the brain showed several acute infarcts in multiple vascular territories with a small associated subarachnoid haemorrhage, consistent with septic emboli. Because of her thrombocytopenia, she was not a candidate for thrombolytics.

**Figure 2:**
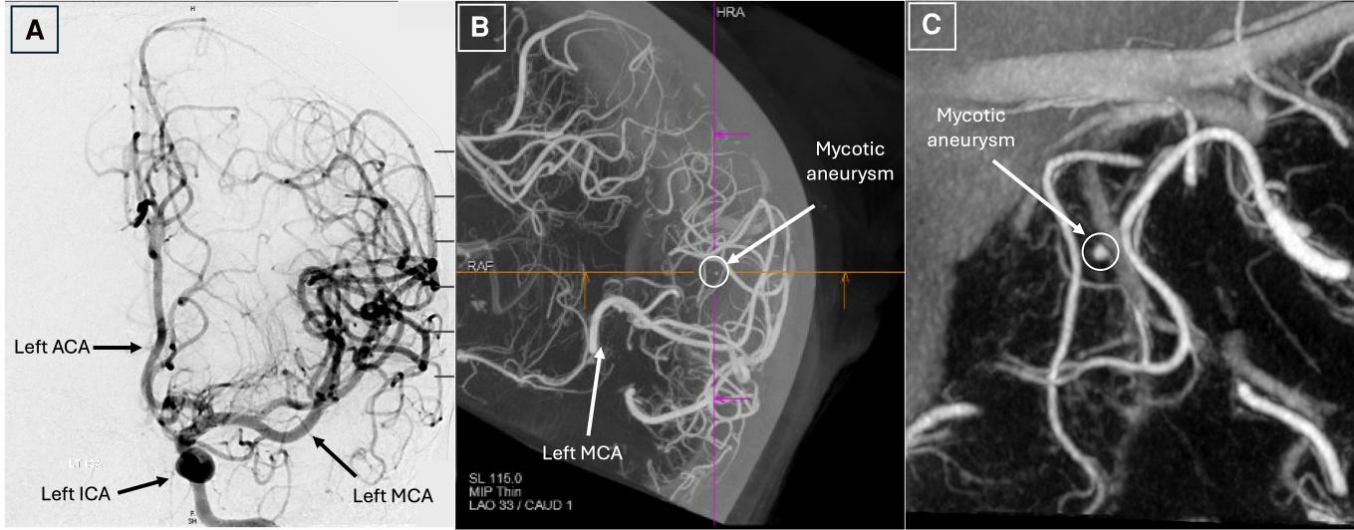
Case 1 (Mycotic Aneurysm): cerebral angiography in coronal plane with Dyna-CT reconstruction sequences. During cerebral angiography, contrast is injected into the internal carotid artery (*Panel A*). Due to the exceedingly small size of mycotic aneurysms, aneurysms are difficult to identify on digital subtraction angiography. However, through Dyna-CT, a technology used to create computed tomography-like images in the angiography suite, smaller vascular abnormalities, like mycotic aneurysms, can be better visualized. The round, protruding opacity seen on DynaCT (*Panels B & C*) represents an aneurysm in the M4 segment of the inferior division of the left middle cerebral artery. In the context of positive blood cultures and infective endocarditis, this is concerning for a mycotic aneurysm. ACA, anterior cerebral artery; ICA, internal carotid artery; MCA, middle cerebral artery.

**Figure 3:**
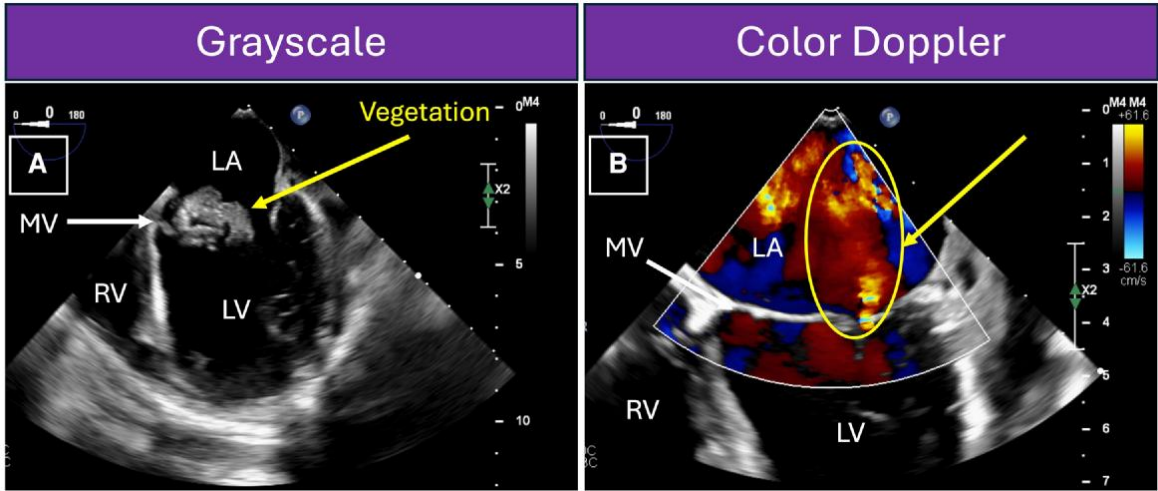
Case 1 (Mitral Valve Vegetation and Mitral Regurgitation): intraoperative transoesophageal echocardiogram (TEE) with midoesophageal two-chamber view at 0° in greyscale and colour Doppler. TEE better visualizes the base of the heart, including the left atrium and mitral valve, providing more precise details regarding the size and location of the mitral valve vegetation. When colour Doppler is placed over the mitral valve (*Panel B*), a red jet is seen in the left atrium, representing blood that is moving towards the probe during systole when the mitral valve closed. This represents mitral regurgitation. This figure corresponds to Supplementary material online, *Videos S3* and *S4*. LA, left atrium; LV, left ventricle; MR, mitral regurgitation; MV, mitral valve; RV, right ventricle.

Urgent surgery was recommended to prevent further embolization and worsening heart failure, given signs of volume overload, a large MV vegetation, and embolic cerebral infarcts. Surgery was delayed while awaiting recovery of thrombocytopenia. Prior to surgery, repeat MRI showed a mycotic aneurysm, which was embolized through a transcatheter approach (*Figure 2*). On day 9 of her hospitalization and at 26 weeks’ gestation, she underwent successful MV replacement with a 27 mm Magna Mitral Ease bioprosthetic valve (Edwards Lifesciences, Irvine, CA) and debridement of a mitral annular abscess, found intraoperatively, without IFM (*Figure 3*; Supplementary material online, *Videos S3–S5*). The surgery required 99 min of normothermic CPB. After surgery, the foetal heart rate tracing was reactive.

Post-operative echocardiogram showed normal left ventricular systolic function and a well-seated and functioning MV prosthesis without paravalvular leaks. Post-operative course was notable for non-sustained ventricular tachycardia. She was discharged on post-operative day 5 with 6 weeks of intravenous ceftriaxone, metoprolol, aspirin, and prophylactic-dose enoxaparin until delivery. She underwent planned caesarean delivery (CD) at 38 weeks without complication. The baby was meeting milestones at 4 months of age. The patient was seen 12 months post-operatively and was doing well.

### Patient 2

**Figure 4:**
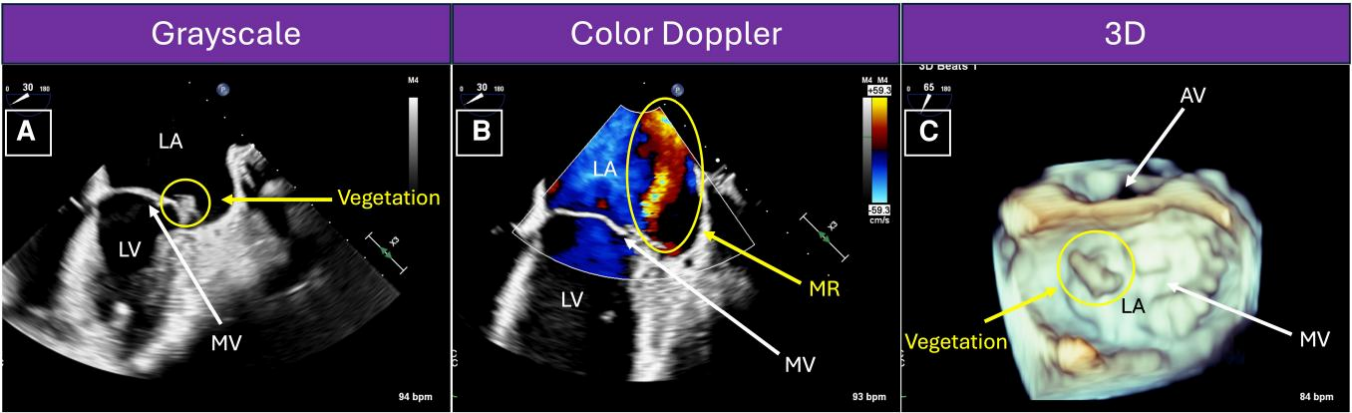
Case 2 (Mitral Valve Vegetation and Mitral Regurgitation): transoesophageal echocardiogram (TEE) with midesophageal two-chamber view at 30° in greyscale and colour Doppler and 3D TEE en face. TEE best visualizes the leaflets and segments of the mitral valve (*Panel A*), allowing for characterization of the vegetation and any related valvular abnormalities. The mitral regurgitation jet seen with colour Doppler (*Panel B*) appears at the exact location of the vegetation, as opposed to the coaptation point of the mitral valve leaflets. This is suggestive of a perforated leaflet. The 3D TEE reconstruction (*Panel C*) in the surgical view visualizes the relationship between vegetation and the other cardiac structures. This figure corresponds to Supplementary material online, *Video S6* and *S7*. AV, aortic valve; LA, left atrium; LV, left ventricle; MR, mitral regurgitation; MV, mitral valve.

A 35-year-old G1P0 female at 15-weeks’ gestation presented with fevers, chills, and malaise. She was noted to be febrile (39.6°C) and tachycardic (122 BPM). Initial evaluation was notable for a positive coronavirus disease (COVID) test. Blood cultures were drawn, and she was diagnosed with a COVID infection and discharged home. One day later, blood cultures grew *Streptococcus mitis*, so she was recalled to the hospital. She was noted to be afebrile (37.1°C) and tachycardic (105 BPM) with a holosystolic murmur. Initial labs were notable for anaemia (10.3 g/dL). She was given intravenous ceftriaxone. Transthoracic echocardiogram showed bileaflet MV prolapse, severe mitral regurgitation, and a 1.1 × 0.7 cm mobile vegetation on the atrial side of the MV. Transoesophageal echocardiogram confirmed bileaflet MV prolapse, severe mitral regurgitation with a flail and perforated A1 segment, and a 1.4 × 0.5 cm mobile vegetation on the atrial side of A1 (*Figure 4*; Supplementary material online, *Videos S6* and *S7*).

**Figure 5:**
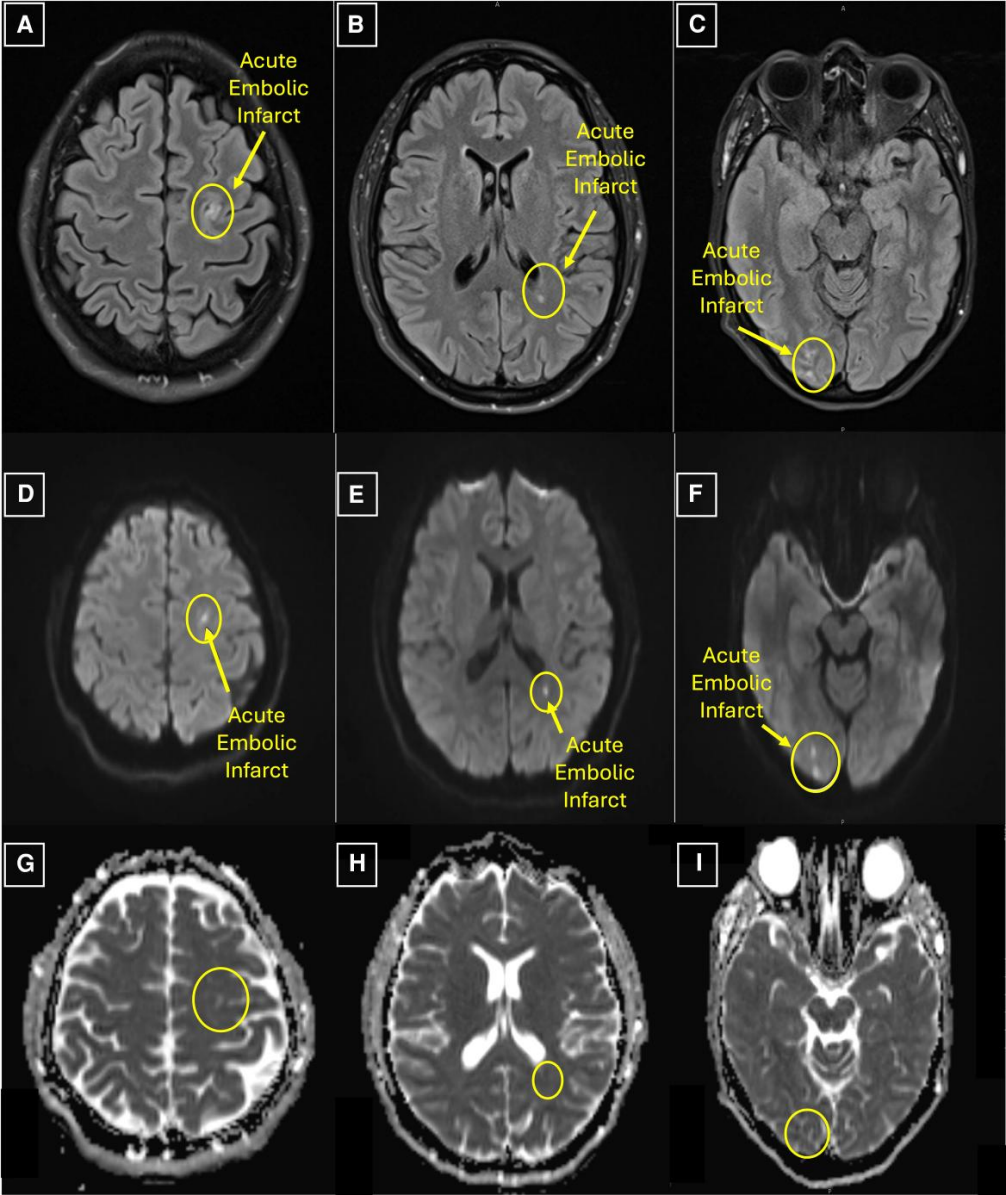
Case 2 (Septic Emboli): magnetic resonance imaging (MRI) in axial view with fluid attenuated inversion recovery (FLAIR) sequencing, diffusion-weighted imaging (DWI), and apparent diffusion coefficient (ADC) sequencing. There are multiple hyperintense foci seen on FLAIR imaging in both cerebral hemispheres at multiple levels throughout the brain (*Panels A, B, &C*). While hyperintensities on FLAIR sequencing are compatible with several diagnoses, they are considered a sign of early stroke. These hyperintense foci are visualized on DWI (*Panels D, E, &F*) but not ADC (*Panels G, H, & I*) sequencing; this pattern of hyperintense foci on FLAIR and DWI without a correlate on ADC is consistent with stroke. In the context of bacteraemia and endocarditis, this confirms a diagnosis of embolic infarcts from septic emboli.

MRI of the brain, obtained to screen for mycotic aneurysms and occult embolic phenomena, revealed embolic infarcts in both cerebral hemispheres without mycotic aneurysms (*Figure 5*). Given the large MV vegetation and embolic cerebral infarcts, the decision was made to proceed to urgent surgical intervention after her blood cultures sterilized to prevent further embolization. On day 5 of her hospitalization, she underwent successful MV replacement with a 31 mm Mosaic mitral bioprosthesis (Medtronic, Galway, Ireland) without IFM. The surgery required 61 min of CPB.

Post-operative echocardiogram showed normal left ventricular systolic function and normal prosthetic MV function. She was discharged with 6 weeks of intravenous ceftriaxone, aspirin, and prophylactic-dose enoxaparin until delivery. She went into labour at 40-weeks’ gestation. She was monitored with telemetry and given ampicillin 1 h prior to delivery. She underwent normal spontaneous vaginal delivery with post-placental copper intrauterine device (IUD) placement. She was seen 7 months post-operatively and was doing well.

## Discussion

We present two cases of IE during the second trimester of pregnancy. In addition to the typical treatment for IE, these cases required multidisciplinary collaboration with a Cardio-Obstetrics team to optimize the care of the patient while minimizing risk to the fetus.^14^ The unique challenges featured in these cases include the timing of delivery, decisions regarding the use of IFM, and the type of valve replacement.

### Relative timing of surgery and delivery

Surgical indications for IE include heart failure, uncontrolled infections, and high risk of embolism.^8^ In both cases, a large vegetation and embolic cerebral infarcts conferred a high embolic risk, and in Case 1, the patient had signs of heart failure. Deferring surgery risked additional embolism and worsening heart failure, so surgery occurred during pregnancy and before the completion of antibiotics, but after improvement in thrombocytopenia (Case 1) and sterilization of blood cultures (Case 2).

For cases of IE during pregnancy, there are three possible sequences of delivery and cardiac surgery: (i) CD followed by cardiac surgery, (ii) cardiac surgery followed by delivery, and (iii) cardiac surgery coinciding with CD. Each sequence creates a unique balance between the risk of CPB to the foetus, namely intraoperative foetal demise, the risk of prematurity and associated complications such as respiratory distress syndrome, developmental delay, and neurologic damage, and the risk of morbidity and mortality to the pregnant patient.^6,15^ By delivering the foetus prior to cardiac surgery, treatment teams can minimize the risk of CPB to the fetus^7^; however, if the gestational age is less than 37 weeks, an earlier delivery may expose the foetus to the risks of prematurity. Alternatively, if cardiac surgery precedes delivery, CPB risks foetal hypoxia, with an estimated one-third probability of foetal mortality,^6,7^ but the foetus can continue to develop *in utero* and, as occurred in both Cases 1 and 2, be delivered at or near full- term.

A combined CD and cardiac surgery was not considered in either case, given the significant intraoperative risk for the patient in Case 1 and the early gestational age of the foetus in Case 2. In Case 1, we recommended proceeding with cardiac surgery without CD to prevent further maternal health deterioration and provide the best chance at optimizing neonatal health by avoiding delivery at an early, periviable gestational age. In Case 2, given the very early gestational age, delivery was not a possibility, and instead the patient was offered termination of pregnancy, which she declined. Ultimately, both patients underwent surgical MV replacement during the second trimester of pregnancy.

### Use of intraoperative foetal monitoring

The decision to pursue IFM incorporates an assessment of foetal viability, the relative risk of the surgery, and the patient’s desire for foetal resuscitation. IFM allows for the titration of CPB settings to improve utero-placental perfusion and permits the possibility of an emergency CD based on signs of foetal distress.^5,16^ However, IFM may not be appropriate when the foetus is previable, the risk of surgery limits the flexibility of titrating CPB settings, an emergent CD during cardiac surgery would pose substantial risk to maternal health, or the patient does not desire foetal resuscitation in the periviable period. The American College of Obstetricians and Gynecologists recommends IFM when (i) the foetus is viable, (ii) IFM is practically feasible, (iii) an obstetrician is available, (iv) the patient has consented to emergency CD, and (v) the planned surgery allows for safe interruption or alteration for emergency delivery.^13^

At 15-weeks’ gestation, the foetus in Case 2 was previable, so IFM was not pursued. Instead, foetal heart tones were recorded before and after surgery.^13^ In contrast, at 25-weeks’ gestation, the foetus in Case 1 was at a periviable gestational age, complicating the decision to pursue IFM. Given the unacceptably high risk of maternal morbidity and mortality if an intraoperative emergency CD were performed or if CPB settings were adjusted to suboptimal levels to accommodate the foetus, we concluded that titrating CPB settings in response to the foetal heart rate tracing risked harming the pregnant patient. Additionally, the patient expressed a desire to optimize foetal maturity. Therefore, through shared decision-making, the decision was made to forego IFM.

### Choice of valve replacement

The final consideration when treating IE during pregnancy involves the choice of valve replacement and anticoagulation. Generally speaking, there are two types of valve replacements: mechanical and bioprosthetic. Mechanical valve replacements are more durable, but carry a higher risk of thrombosis, requiring anticoagulation with warfarin; bioprosthetic valves do not require anticoagulation but eventually degenerate. Although there is some evidence to suggest that bioprosthetic valves are durable and effective for younger patients,^17^ professional society guidelines state it is reasonable to pursue mechanical valve replacements in younger or middle-aged patients.^10,11^ However, mechanical valves require anticoagulation with warfarin, which is teratogenic and carries a higher risk of maternal bleeding. Data from the Registry of Pregnancy and Cardiac disease shows a 4.7% risk of thrombosis and a 23.1% risk of haemorrhage in patients with mechanical heart valves,^18^ necessitating careful decision-making regarding the choice of valve replacement and anticoagulation during pregnancy.

Since valve repair was not feasible in either case, both patients chose between types of valve replacements. In Case 1, a bioprosthetic valve was favoured given the elevated risk of intracranial bleeding after recent stroke. After extensive counselling, both patients elected bioprosthetic valve replacements. While both patients avoided the need for warfarin and were instead treated with aspirin and enoxaparin, they accepted the risk of valve deterioration and need for a future valve replacement. Again, both cases highlight the importance of shared decision-making regarding valve choices.

This series highlights the unique pregnancy-related considerations of caring for patients with IE, which require the involvement of multidisciplinary teams to make individualized decisions regarding the relative timing of delivery and cardiac surgery, the use of IFM, and the type of valve replacement. These decisions should always be made in collaboration with patients to ensure that patients’ values and preferences are incorporated into the treatment plan.

## Lead author biography


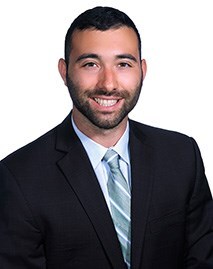


Jordan is an internal medicine resident at NYU Grossman School of Medicine. In addition to a medical degree, Jordan also holds a master’s degree in bioethics. His primary research interests include transplant ethics, decision-making for left ventricular assist devices, and improving medical ethics education. He has contributed to research studying brain-death, multimodal cardiac imaging, and normothermic regional perfusion, as well as working groups on paediatric gene therapy and xenotransplantation. He plans to continue his training as a cardiologist, where he hopes to apply his expertise in medical ethics and continue to improve the care of vulnerable patients.

## Supplementary Material

ytag057_Supplementary_Data

## Data Availability

The data underlying this article are available in the article and in its online Supplementary material.
